# Infrasound detection of approaching lahars

**DOI:** 10.1038/s41598-023-32109-2

**Published:** 2023-04-20

**Authors:** J. B. Johnson, A. Roca, A. Pineda, R. Mérida, R. Escobar-Wolf, J. F. Anderson, J. Mock, A. Bosa, G. Bejar, G. P. Waite

**Affiliations:** 1 Department of Geosciences, Boise State University, Boise, USA; 2 Instituto Nacional de SismologíaVulcanologíaMeteorología e Hidrología (INSIVUMEH), Guatemala City, Guatemala; 3 Unaffiliated, Guatemala City, Guatemala; 4 Department of Geological and Mining Engineering and Sciences, Michigan Technological University, Houghton, USA

**Keywords:** Natural hazards, Hydrology

## Abstract

Infrasound may be used to detect the approach of hazardous volcanic mudflows, known as lahars, tens of minutes before their flow fronts arrive. We have analyzed signals from more than 20 secondary lahars caused by precipitation events at Fuego Volcano during Guatemala’s rainy season in May through October of 2022. We are able to quantify the capabilities of infrasound monitoring through comparison with seismic data, time lapse camera imagery, and high-resolution video of a well-recorded event on August 17. We determine that infrasound sensors, deployed adjacent to the lahar path and in small-aperture (10 s of meters) arrays, are particularly sensitive to remote detection of lahars, including small-sized events, at distances of at least 5 km. At Fuego Volcano these detections could be used to provide timely alerts of up to 30 min before lahars arrive at a downstream monitoring site, such as in the frequently impacted Ceniza drainage. We propose that continuous infrasound monitoring, from locations adjacent to a drainage, may complement seismic monitoring and serve as a valuable tool to help identify approaching hazards. On the other hand, infrasound arrays located a kilometer or more from the lahar path can be effectively used to track a lahar’s progression.

## Introduction

### Lahar hazards and monitoring

In terms of number of lives lost, lahars are the most impactful of volcanic hazards over the last century. For example, the 1985 eruption of Nevado del Ruiz (Colombia) killed between 23,000 and 26,000 people when volcanic mudflows reached the flat valleys where the cities of Armero and Chinchina were located^1,2^. The catastrophe was particularly devastating given that the lahars originated near the summit and more than two hours before their arrival at Armero at about 70 to 75 km distance^3–5^. Functional early warning systems might have provided an opportunity for local communities to evacuate. In the wake of the Armero tragedy, lahar hazards have been recognized at many active, as well as dormant and extinct volcanoes. Many volcanoes in the Cascades Range, for example, have significant lahar hazards including Mount Rainier^6^ where large pre-historic flows occurring 500 and 5600 years ago have inundated currently heavily populated areas^7^. As of 2009, more than 78,000 people resided in Rainier’s lahar hazard zones^8^. Recognizing this risk, monitoring efforts by the United States Geological Survey have been implemented to integrate seismic and infrasonic early warning systems^9^. Integrated seismo-acoustic monitoring has previously been used elsewhere, for example in the European Alps to identify and monitor debris flows in non-volcanic regions^10^.

Geophysical monitoring at volcanoes is pertinent for eruption forecasting and detection of both syn-eruptive hazards (e.g., primary lahars) as well as surveillance of hazards unassociated with eruption (e.g., secondary lahars)^11^. Efforts to monitor lahar activity have traditionally focused on relatively high frequency (above 10 Hz) seismic measurements using geophones, referred to as acoustic flow monitors (AFMs^12^); detection of ground vibrations associated with passing mass movements have proven valuable for identifying the occurrence, duration, and relative size of mudflows and debris flows (e.g.^13–17^). Seismic monitoring of lahar channels at Fuego Volcano, using both short-period (above 1 Hz) and broadband seismometers, has already been implemented by the Guatemalan monitoring agency, the Instituto Nacional de Sismología, Vulcanología, Meteorología e Hidrología (INSIVUMEH; https://insivumeh.gob.gt/), to issue timely public bulletins of lahar activity. Their telemetered monitoring network permits near-real-time notice of lahars generally issued within tens of minutes of a flow’s passage at seismic sites, which at Fuego are situated near several drainages approximately 10 to 15 km from the summit. Flow occurrence is subsequently confirmed by first-hand observations or telemetered webcams. In general, high-frequency seismic waves produced by lahars appear to attenuate rapidly, which is why seismic lahar monitoring at Fuego has been installed adjacent to potential lahar channels and are particularly sensitive to local lahars as they pass by.

Lahars are known to advance at a range of speeds from a few meters per second (e.g.^18–20^) to several tens of meters per second (e.g.^21,22^), depending upon their size, composition (blocky, concentrated, or non-concentrated), and channel morphology (width, tortuosity and gradient)^3^. Given these velocities and location of lahar initiation zones, which is often high up on a volcano, timely alerts from AFMs and seismometers could be made reliably using upstream sensors to notify downstream communities of an approaching flow. This is not easily done at Fuego and other volcanoes, however, because logistics, access issues, and site maintenance are complicated higher up on the volcano. At Fuego Volcano, moderate-sized secondary lahars reaching populated areas (generally beyond 10 km) appear to travel with speeds of 4–8 m/s (^18^, this study). These relatively slow speeds imply that about two to four minutes of advance notice could be possible for each km of flow distance.

Our study of lahars is predicated on the concept that infrasound monitoring may complement seismic monitoring because it is capable of detecting the flows from a distance of many kilometers (e.g.^22^) and could thus be used to provide time sensitive alerts. Although seismic networks have demonstrated some potential for tracking distant lahar movements^23^, infrasound arrays have been shown to be particularly effective for identifying and tracking mass movements for a wide range of geophysical moving sources, including debris flows^24^, icefall and rockfall^25,26^, snow avalanches^27^, and pyroclastic density currents^28^. Here we compare lahar seismic signals with *‘broadband sound’* defined as extending from the near-infrasound band (1–20 Hz) into the low frequency audible band above 20 Hz. This study uses visual observations of flow characteristics, including timing and size, integrated with infrasound for a well-monitored event on August 17 using seismo-acoustic equipment and multiple cameras filming the flows. Following the analysis of this well-monitored flow, we then show that remote detection of relatively small mudflows using a single infrasound site is practical for an entire season of lahar activity using a catalog of more than 20 events from 2022.

We use Fuego Volcano (Guatemala) as a natural laboratory for lahar study given that it frequently produces rainy season lahars, particularly after eruptions that leave pyroclastic flow deposits within its active channels^29^. Typical Fuego eruptive activity includes small Strombolian to ash-rich eruptions, which deposit ash and tephra on the upper slopes, as well as sporadic larger eruptions, which emplace larger quantities of pyroclastic flow material in valley drainages during hours-to-days long paroxysms^30,31,34^. Recent paroxysms at Fuego include the disastrous June 3, 2018 event, which buried the community of San Miguel Los Lotes^35^, as well as more recent, but smaller, paroxysms occurring on March 7 and July 4, 2022. Ample material from the sporadic larger eruptions is available as a ready source of mud and hot debris, which contribute to the bulking materials for the frequent lahars occurring during Guatemala’s rainy season. Between June and October intense thunderstorms and rain events are common at Fuego and induce repeated secondary lahars similar to the activity observed at other frequently erupting monsoonal volcanoes, such as Merapi (Indonesia) and Colima (Mexico)^32,33^.

### Experiment at Fuego volcano

**Figure 1:**
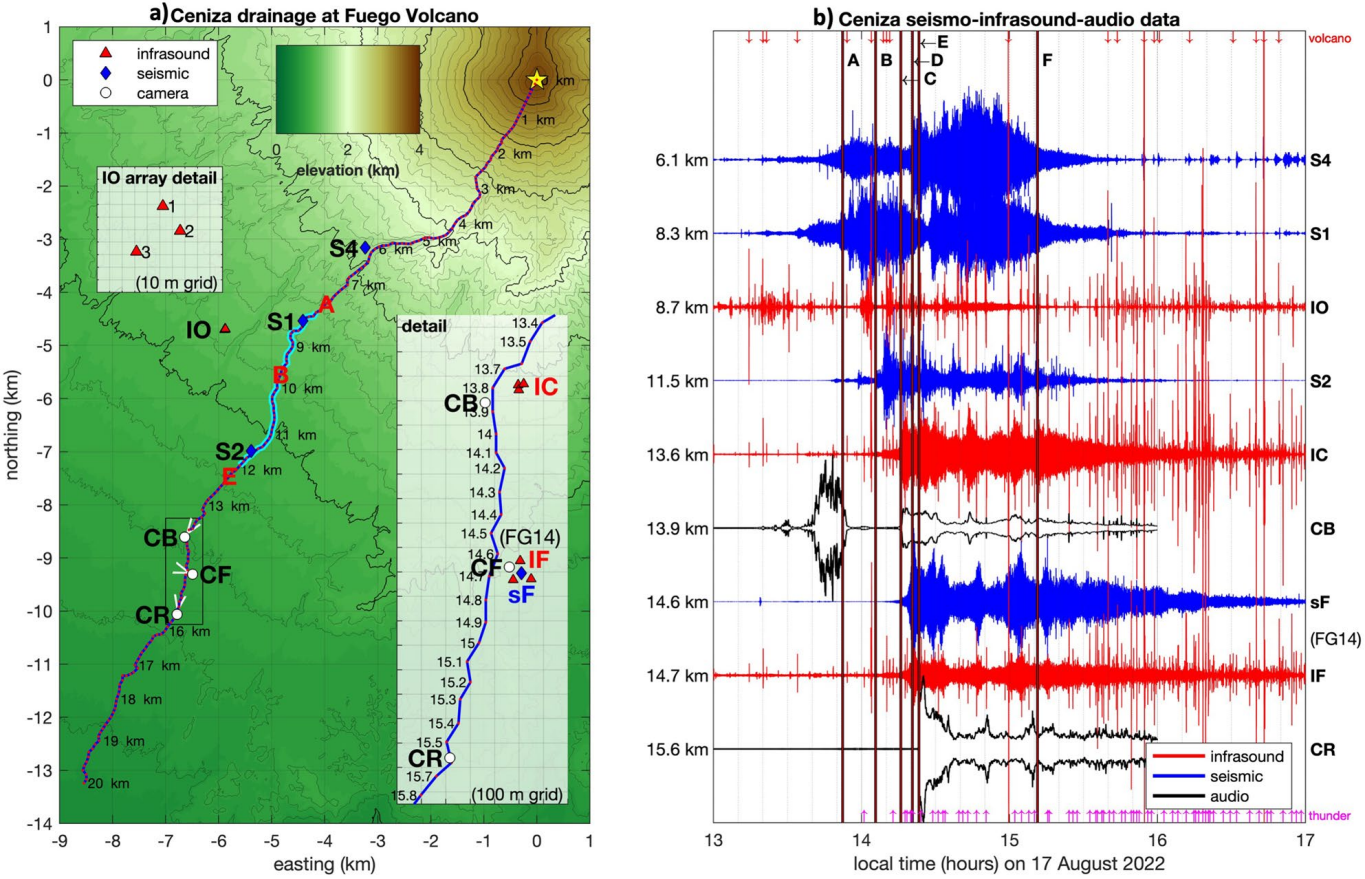
(**a**) Map created with Matlab software of Ceniza drainage on the SSW side of Fuego Volcano showing locations of seismic, infrasound, and camera locations during the 2022 season. Channel distances, relative to summit, are indicated. Inset maps show detail of IO array and the instrumentation configuration near the three cameras. The cyan-highlighted section of Ceniza channel shows regions where station IO is most sensitive to the moving lahar source; locations A, B, and E correspond to featured timing of IO infrasound detections. (**b**) Normalized waveforms from August 17 show vertical seismic channels, a single microphone channel from each of the three infrasound arrays, and acoustic power extracted from the audio microphone of cameras CB and CR. Vertical time lines A–F are described in the text and in Table 2. Vertical magenta and red arrows correspond to infrasound detections of thunder and volcanic event activity respectively.

Secondary lahars at Fuego, induced by rainfall, may be reliably observed during targeted short-duration field campaigns, which use proximal (< 100 m from lahar channel) sensors and distant (more than 1 km from channel) infrasound sensor arrays, seismic monitoring apparatus near the drainage, time lapse cameras, and time-synchronized video cameras and their audio channels. Most large drainages radiating from Fuego are active lahar paths; however, our 2022 experiment focused on the Ceniza drainage on the SSW side of the volcano (Fig. 1a) as it has been the most active channel during previous years, with dozens of events typically occurring during the four-month rainy season. Other drainages radiating from Fuego are also active lahar paths (e.g., Las Lajas^18^), however they are monitored locally and their activity is not readily detected by the Ceniza instrumentation.

**Figure 2:**
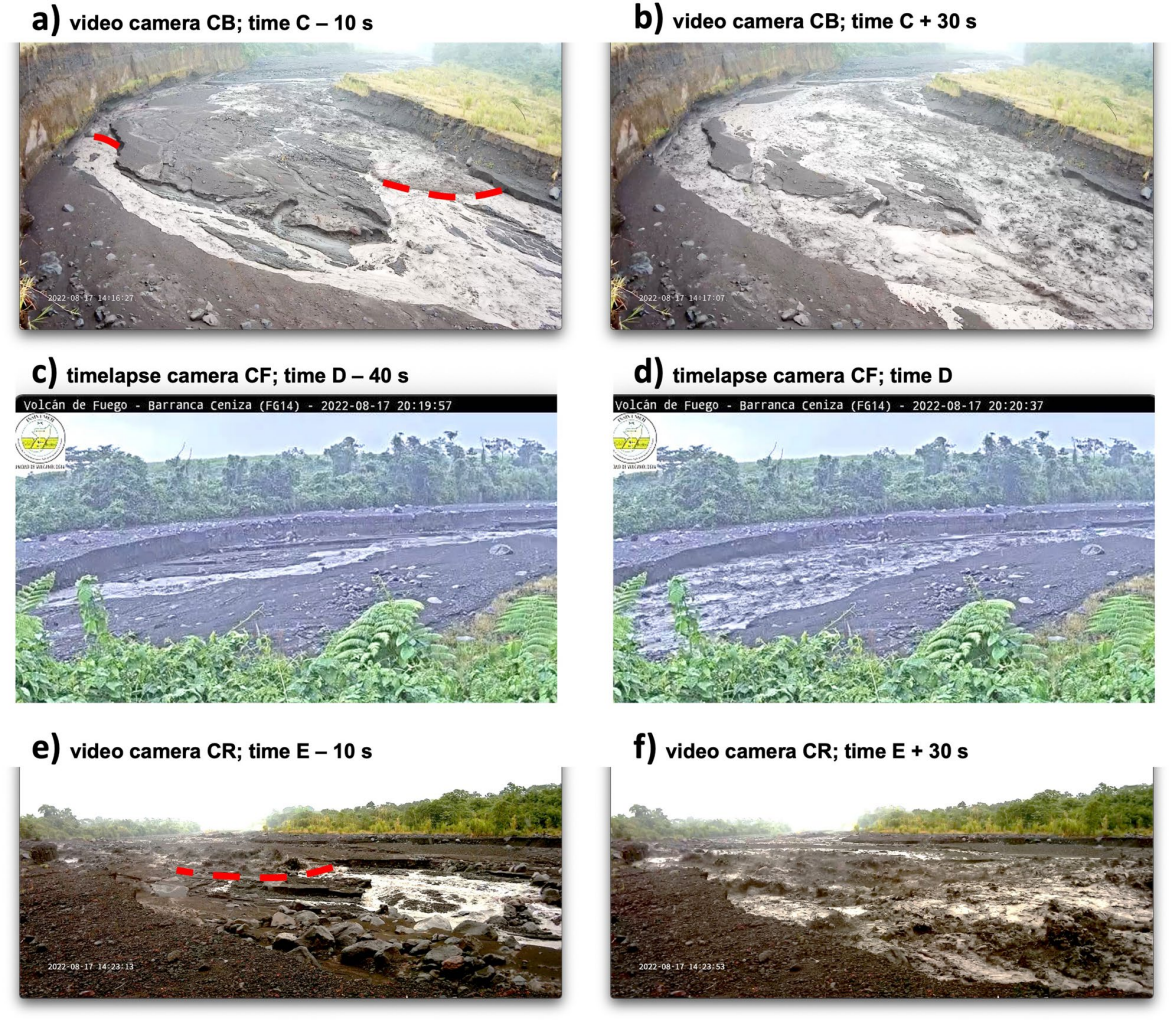
Video still images corresponding to (**a,b**) video camera at CB around time C, (**c,d**) time lapse camera at CF around time D, and (**e,f**) video camera at CR around time E. In each case still frames are provided with 40-s separation bracketing the arrival time of the lahar at those camera locations. In panels (**a,e**) the approaching flow front is annotated as a dashed red line for visibility. These images were extracted from cameras deployed during the field campaign.

Our experiment targeted Ceniza with instruments situated 6 to 16 km from the summit along the stream channel and included both campaign-style, short-term sensor deployments as well as data from the continuous long-term monitoring site known as FG14 (at 14.7 km channel distance from Fuego). The telemetered FG14 station is on the edge of Ceniza drainage and is operated by INSIVUMEH; in 2022 it consisted of a time lapse camera (CF), a 100-m aperture infrasound array (IF), and a short period vertical component seismometer (sF). Temporary stand-alone stations S4, S1, and S2 were broadband triaxial seismometers, while stations IO and IC were ~ 30-m-aperture, 3-element infrasound arrays with flat response between 0.1 and 100 Hz. All stations and sensors were located within 100 m of the channel except for station IO at the Fuego Observatory (1.3 km from the Ceniza channel’s closest approach). The highest concentration of sensors and cameras was situated between 13.5 and 16 km channel distance (inset in Fig. 1a) and included both INSIVUMEH’s time lapse camera CF at FG14 (recording at 40-s intervals) as well as two wide-angle high-definition video cameras at CB and CR recording at 25 frames per second (Fig. 2; Table 1).

**Table 1:** Description of sensors and their distances along the channel along with deployment comments.

Name	Channel distance	Sensor type	Comment
S4	6.1	Broadband seismometer	Edge of Ceniza
S1	8.3	Broadband seismometer	Edge of Ceniza
IO	8.7	3-element infrasound array	Situated 1.3 km from Ceniza
S2	11.5	Broadband seismometer	Edge of Ceniza
IC	13.6	3-element infrasound array	Edge of Ceniza
CB	13.9	Video camera	Audio channel available
sF	14.6	Short period vertical seismometer	Part of FG14 station (INSIVUMEH)
IF	14.7	4-element infrasound array	Part of FG14 station (INSIVUMEH)
CF	14.7	Time lapse camera (40 s)	Part of FG14 station (INSIVUMEH)
CR	15.6	Video camera	Audio channel available

The Ceniza drainage near FG14 (at 12 to 16 km) is usually a nearly dry flat-bottomed wash (~ 30–50 m wide) with ankle-to-knee-deep slow-moving water in braided channels (3–5 m wide); however, this stream can change rapidly and dramatically during precipitation events, which occur during common thunderstorms from May through October and often in the late afternoon. Between April 19 and October 13, 2022, at least 35 lahars occurring in Ceniza were reported in bulletins by INSIVUMEH of which approximately 22 occurred during daylight hours and with good webcam observations. These events’ sizes are determined by local observers who combine visual assessment of width and depth of flow, size and number of blocks transported, as well as amplitude and duration of seismic amplitudes at station sF (FG14). During the daylight hours in the 2022 season 14 Ceniza events were characterized as *moderate-to-strong *(*fuerte*), 4 as *moderate *(*moderado*)*,* 14 as *weak-to-moderate,* and 3 as *weak *(*debil*). These lahar sizes are assessed qualitatively by INSIVUMEH analysists who ranked their size primarily based on seismic amplitudes at sF rather than by the visual observations. The largest of these flows occupy much of Ceniza’s channel when they pass the timelapse camera at CF at a distance of 14.7 km.

We organized our field campaign and sensor deployment in late August 2022 to coincide with anticipated intense lahar activity and had the expectation of approximately two events occurring each week. Our full suite of monitoring instrumentation was in place at Ceniza before August 17 (Fig. 1b) when a high quality, rain-induced event occurred that was ranked by INSIVUMEH staff as *moderate*. We first analyzed the event with the three-element infrasound array (IO) located at Fuego’s observatory to detect and track coherent sound in the 1 to 100 Hz filtered sound band. Our strategy for this distant array was to identify a moving source and track its progression using signal cross-correlation analysis with overlapping 5-s time windows and 1-s time steps (e.g.^22,36^**,** “Methods”). This event was relatively easy to analyze because thunderstorm activity, which can produce high-amplitude transients that can obscure lahar infrasound signal^37^, was relatively minor on August 17.

Using the onset of sustained highly correlated signal as a detection, the first indications of a lahar at IO was at 13:52 local time (19:52 UTC) (time A in Fig. 1b) and coincides with sound originating from Ceniza between channel distance 8 and 9 km. Its source within the Ceniza drainage is located by matching the expected infrasound time lags, for all candidate locations within the channel, with cross-correlation lag times calculated from the infrasound data recorded by the array (refer to section on “Methods” for more details). Evolving source locations, notable event times, and transitions in the inferred location within Ceniza are summarized in Table 2. For example, at 14:06 (time B) the source of infrasound appeared to migrate downstream towards a region around 11 to 12 km. At times C, D, and E the lahar successively crosses the field-of-view of cameras CB, CF, then CR after which infrasound source detection returns to the region between 8 and 9 km. We note that infrasound signal amplitude and coherence taper by 15:11 (time F) about 80 min after first lahar signal detection. Following time F the detectable infrasound from downstream reaches of Ceniza’s channel remains apparent for several hours. We attribute this non-lahar acoustic signal to turbulent stream features and concentrated flow, which has been shown to radiate infrasound in fluvial environments (e.g.^38^).

**Table 2:** Description of times indicated in figures.

Time code	Time on 17 August	Comment
A	13:42	First detection of lahar infrasound at IO
B	14:06	Detection of movement below 10 km channel distance
C	14:16	Flow seen passing CB (adjacent to IC)
D	14:21	Flow seen passing CF (at FG14)
E	14:23	Flow seen passing CR
F	15:11	Infrasound signal coherence drops at IO

Time elapsed between flow front arrivals and distance difference between the C and E cameras are used to compute an average flow speed of 4.2 m/s for the initial lahar head for the August 17 event. This corresponds to a 7.1-min transit time for the 1.8 km channel transect. Average speed in this section of the channel appears relatively slow compared to some published lahar speeds (e.g.^21,22^), however it is in line with cited values from smaller secondary lahars elsewhere at Fuego^18^ and in some other studies where secondary lahars are common, e.g., at Colima and Popocatepetl^16,19^**.** The slow flow speed may be attributed to the relatively low gradient between cameras CB and CR, which drops less than 100 m per km.

## Results

### Interpretation of Lahar infrasound

**Figure 3:**
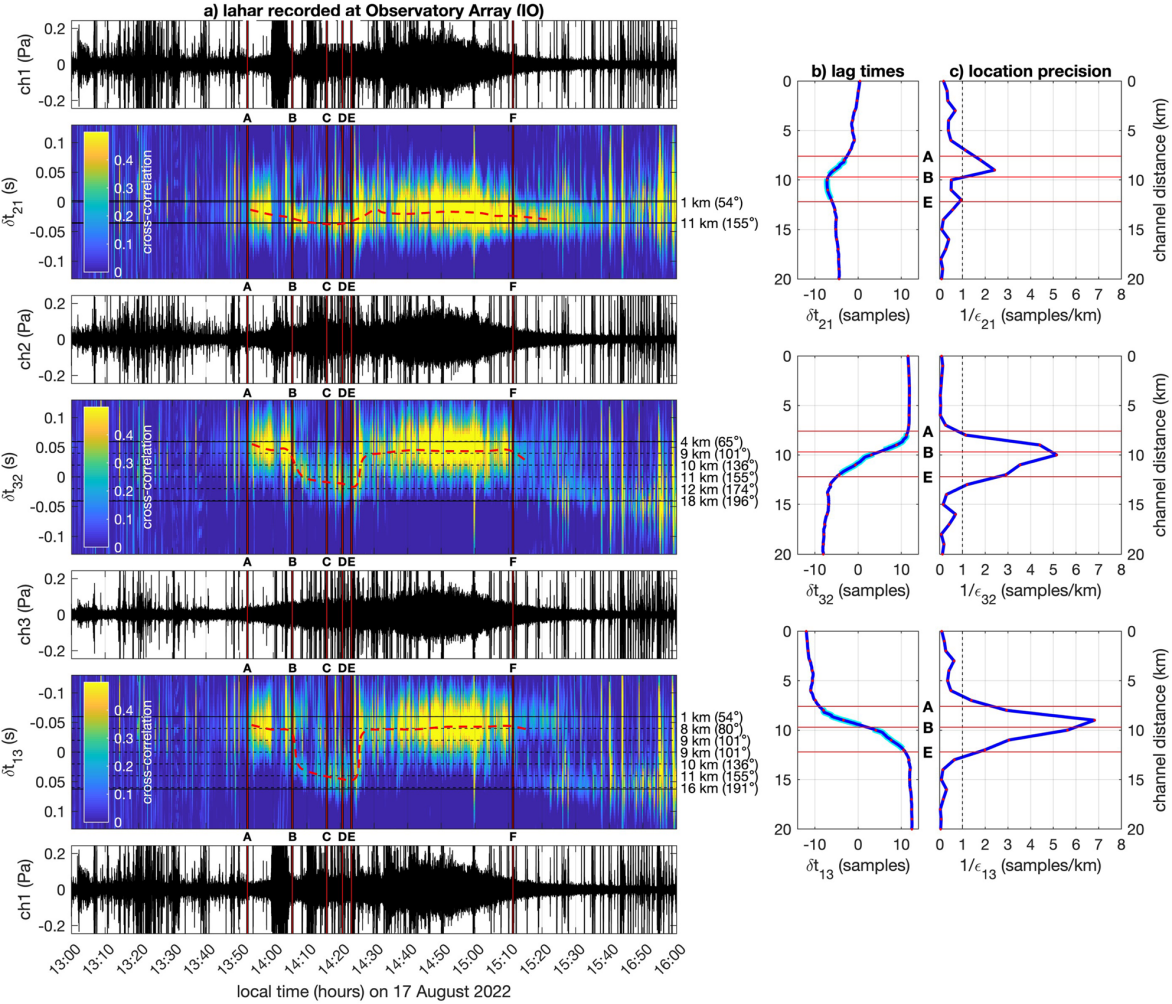
Cross correlation analysis from station IO is used to detect sound originating from the Ceniza drainage. (**a**) Waveforms are shown in black whereas time lag evolution between microphone channels are indicated with colormaps of normalized cross-correlation values as a function of time and time lag. Changing lag times indicate moving sources. The lefthand ordinate indicates lag time, whereas the right ordinate corresponds to projection of those lag times on to a position within Ceniza’s channel (refer to panel **b**) and parenthetical azimuthal directions. Yellow hot spots starting at 13:52 (time A) indicate first detection of lahar; other times (B–F) are explained in Table 2. Red dashed lines indicate consistent sources and peak cross-correlation time lags associated with lahar activity. (**b**) Lag times, or phase delays between sensor pairs, are indicated assuming sources confined within the Ceniza drainage. (**c**) Source precision, calculated as the inverse of source uncertainty (ε), is in units of samples per kilometer. Values to the right of the dashed line indicate regions along the channel path where location resolution is best, i.e. positional change of 1 km gives rise to a change of more than 1 sample of cross-correlation timing difference.

Lahar infrasound signals are long in duration and appear tremor-like, emerging gradually and decaying even more slowly (e.g.^22^.). Infrasound recordings from the featured lahar on August 17 appear ‘noisy’ at IO in the sense that the signal is punctuated by high amplitude short-duration pulses, which correspond to two types of transient events including: (1) repeated thunder, which is a spatially-distributed broadband source (e.g.^37,39^.), and (2) explosive Strombolian activity, which is sourced at Fuego’s vent and has a median frequency value less than 10 Hz (e.g.^40^). These volcano and thunder signals can ‘contaminate’ lahar signal and impede signal detection, however they can also be identified and excluded given their high-amplitude, short-duration transient nature (refer to Fig. 1b and discussion of thunder and explosion classification in “Methods”). Visual review of the cross-correlation analyses is particularly effective as a tool to identify the lower amplitude tremor-like lahar signals because correlations from these flow signals are extended in time duration and have either gradually-varying or static time lags (see red dashed lines in Fig. 3a). Cross-correlation signal detection is a powerful tool even when infrasound is obscured by incoherent wind noise, which was significant at station IO, where two of three sensors had to be located on the observatory rooftop.

The observatory infrasound array IO, which is situated 1.3 km from Ceniza’s closest approach, is used primarily to identify the lahar onset and to track the movement of an initial energetic flow pulse, which is a common feature for many lahars manifested as a *head* wave (e.g.^20,41^.). We note however that once the initial surge propagates sufficiently far downstream the IO array becomes preferentially sensitive to proximal sources occurring in the closest section of the channel. This is most notable as the August 17 flow begins to ebb (shortly after time E) and the primary infrasound source location appears to return to 9 to 10 km along the channel. One of the challenges of tracking lahars, or other types of mass movements (e.g., rockfalls, debris flows, or pyroclastic density currents^42^), is that sound sources are distributed over large spatial extents and are long-duration lasting tens of seconds to hours. Their non-compact sources are dynamic in size and source position such that different stations may be sensitive to different sections of a flow path. Another challenge is that the lahar generation process is non-instantaneous and may occur over an extended spatial region producing multiple flow surges^18^, which may or may not coalesce^43^.

**Figure 4:**
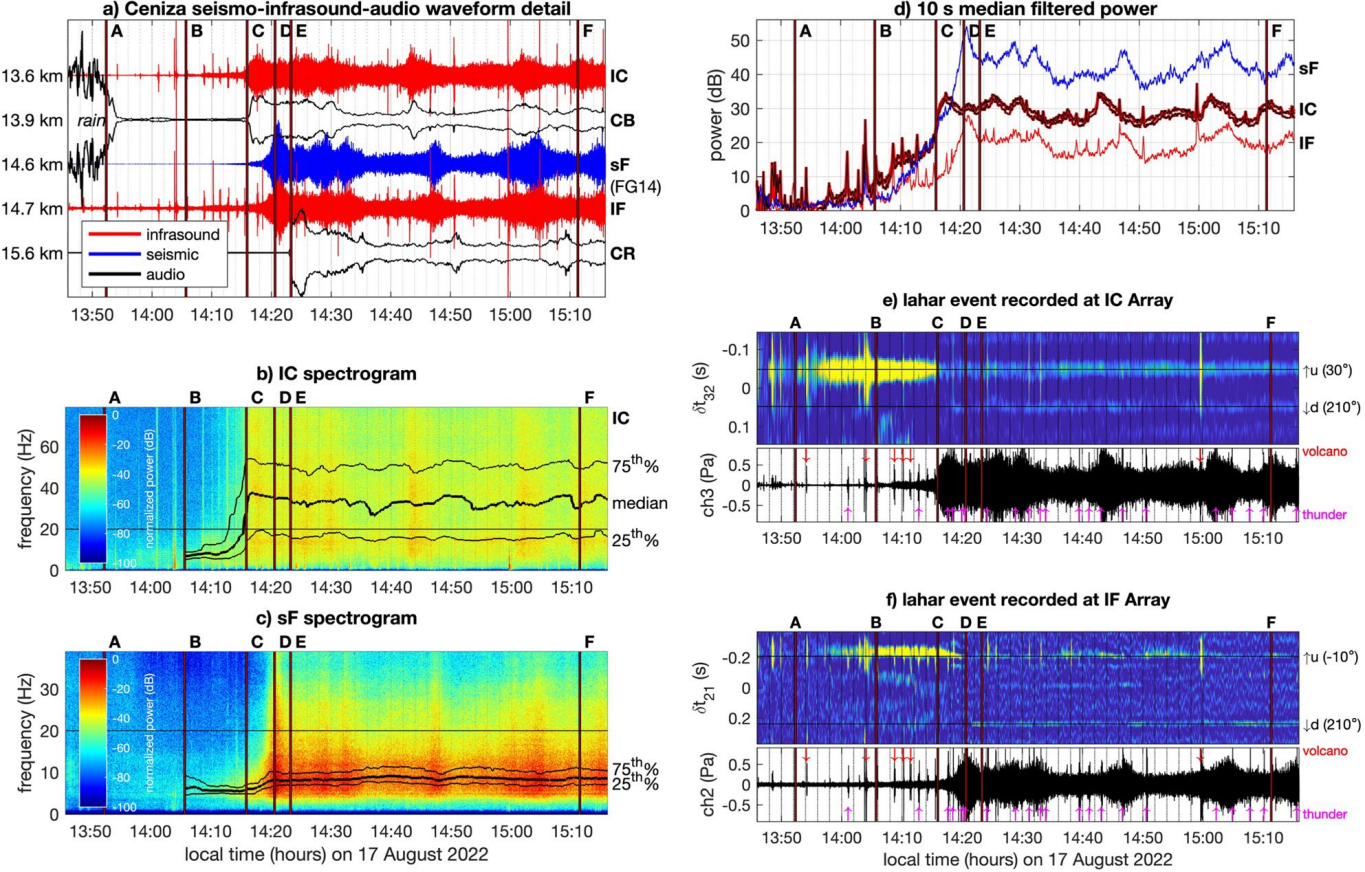
90-min time series from August 17, 2022 showing (**a**) detail of waveforms from IC, CB, sF, IF, and CR and (**b,c**) spectrograms from IC infrasound and sF seismometer. (**d**) Infrasound from stations IC and IF and seismic from sF is converted to power and displayed logarithmically to show emergent lahar signal. (**e,f**) Cross correlation analyses from station pairs at IC and IF show about 22 min of precursory infrasound prior to lahar’s arrival at cameras CB and CF respectively. Up and down black arrows indicate correlated energy arriving from simultaneous upstream (u) and downstream (d) sources after the flow front has passed. Magenta and red arrows denote times of detected thunder and volcano sources.

Infrasound detections from arrays located immediately adjacent to the Ceniza drainage at IC and IF show different patterns of lag time evolution compared to IO (Figs. 3a, 4e,f) and they can not generally be used to pinpoint a sound source position within the channel. They can, however, be used to detect lahar sources originating both upstream and/or downstream. For example, both the IC and IF arrays indicate strongly correlated sound beginning soon after time A. This strong signal correlation ends abruptly at times C and D when the flow front reaches the cameras that are co-located with the respective arrays. Thereafter, faint coincident dual sources become evident in the correlation analysis pointing to signals originating from both upstream and downstream azimuths. Coherent sources are detected because these distributed regions of the drainage are filled with flowing debris creating a superposition of sound, which share a common incidence. Because the river geometry is nearly straight for long segments there tends to be strong detections originating from the two distinct directions. In contrast, those acoustic sources in the river adjacent to the arrays do not produce coherent signal because they bombard the array from a wide range of azimuths. This observation is relevant for informing the situation of near-channel infrasound monitoring arrays. Tracking of moving flow sources is generally improved if the array is deployed more than a few hundred meters from the channel and if the array aperture is small relative to the distance between array and lahar source (e.g.^18^.). Array IO, for instance, is effective for tracking a moving flow source, whereas arrays IC and IF may be better suited for early detections.

The early detection of the lahar with both IC and IF is notable and testament to the remote sensing capabilities of an infrasound monitoring system that is situated at river’s edge and below an extended section of relatively straight channel. This array topology permits detection of 22 min of very clear precursory infrasound prior to the flow’s physical arrival for the August 17 event. Assuming that the flow front advances no slower than 4.2 m/s (and likely faster higher up on the volcano) a 22-min precursor implies that the initial lahar signal detection is associated with a source at least 5.5 km upstream. This coincides with an origination zone less than 8 to 9 km channel distance and in the region where IO first picks up the moving source at time A. This section of the channel also coincides with the inferred depositional region for the July 4, 2022 pyroclastic density current, which likely served as an important source of material that could have contributed to bank collapse and rapid flow bulking. Hot debris is evidenced by the steaming mud and blocks that are visually evident in video about five minutes after the initial flow’s arrival.

### Seismic and broadband acoustics

The 22-min lahar sound precursor is dominated by acoustic energy concentrated in the infrasound band, but its frequency content increases noticeably as the flow approaches (Fig. 4b). At around 3 min prior to the flow’s arrival the median frequency increases and crosses into the audible range (above 20 Hz). This frequency shift coincides with an inferred flow front located about 1 km upstream from the monitoring array. Once the flow has arrived the median frequency remains relatively constant at 30 to 35 Hz. Notably, the audible-band sound extracted from cameras CB and CR is only evident as the flow arrives and afterwards (Fig. 4a). Although Fuego observatory staff report that sounds originating from an approaching lahar are common, we conclude that the audible warning precursor is likely only useful as a few tens of seconds warning. Our spectral observations point definitively to the benefit of broadband sound monitoring and early detections of lahars using the infrasound band.

As with infrasound, the seismic spectral content (Fig. 4c) also shows a similar, albeit less pronounced, rising evolution in its frequency content beginning several minutes prior to the arrival of the lahar. Between times C and E the median spectral content of the seismic tremor increases from 6 to 8 Hz and then remains relatively constant. At present we are unable to determine whether the seismic signal is dominated by ground-propagating seismic waves, air-to-ground coupled sound, or a combined-influence seismo-acoustic wavefield.

Both seismic and infrasound channels indicate precursory wave energy reaching IC, IF, and sF well prior to the lahar’s arrival (Fig. 4d). In both infrasound and seismic wavefields the signals are emergent and hard to discern by viewing a linearly scaled pressure or velocity time series; the precursors become more evident following conversion of the waveform signals to power, removal of high amplitude transients (thunder and explosions) using median filters, and displaying in decibels or using logarithmic axes. For the August 17 event, both infrasound and seismic power are identifiable by about 14:00, which is at least 15 min prior to the arrival of the flow at station IF and sF and only a few minutes after cross correlation analysis initial detections (Fig. 4e,f). Following the flow’s arrival there are fluctuations in seismo-acoustic power evident in the logarithmically-scaled displays (Fig. 4d), which are likely associated with oscillating flow levels or small surges similar to those described at Fuego by^18^. For the August 17 event these pulses correspond to relatively small surges lasting only a few minutes and are evident in the videos from CB and CR (viewable at https://www.youtube.com/watch?v=O9lJT52MVik and at data repository—see Data availability).

## Discussion

### Ensemble analysis and utility of infrasound for early warning of Lahars

With lessons learned from the August 17 lahar, we analyzed 22 events occurring in 2022 that were well recorded with the telemetered station FG14 (i.e., sF, IF, and CF) and occurred during daylight hours (Table 3). We performed the same correlation analysis on channels 1 and 3 of array IF that was done in Fig. 4f to highlight the monitoring capabilities of using just two microphones for detections. We then used timelapse video to identify an initial lahar flow surge, which is a common feature of most of Fuego’s secondary lahars in Ceniza. We note that for those events where streamflow is already elevated a definitive lahar flow front surge is sometimes less apparent. INSIVUMEH recognizes these events, which begin gradually in terms of their seismic signature, as *correntadas*, or currents. Visually, they begin as river flows and do not initially convey large bulky bed load.

**Table 3:** Summary of 2022 Ceniza lahar statistics (see supplementary animation).

Event date and time	Video surge detection (*)	INSIVUMEH report time	INSIVUMEH size (video size)	Infrasound precursor time (min)	Comment
27 May	14:42	14:50	M/S (3)	14:12 (30)	Strong flow front; long precursor
06 Jun	13:30	13:40	W/M (2)	13:18 (12)	Strong pulse at flow front; good precursor
13 Jun	16:15	16:00	W/M (1)	16:13 (2)	Precursor hidden by thunderstorm noise
20 Jun	16:05	16:00	M/S (1)	15:36 (29)	Pulsed precursor and pulsed flow
21 Jun	15:06	15:20	M/S (2)	14:56 (10)	Pulsed flow weakly evident upstream
12 Aug	17:07	17:10	M/S (1)	Na	Very small; precursor hidden by thunder
17 Aug	14:20	14:30	M (2)	13:58 (22)	Strong obvious flow arrival and good infrasound precursor
24 Aug	13:42*	13:45	M/S (1)	Na	INSIVUMEH alert was made relatively early and during *correntada*. An obvious surge is evident at 14:08 with a ~ 8 min infrasound precursor
25 Aug	16:15	16:15	M/S (3)	15:46 (29)	Excellent long infrasound precursor; strong flow front
29 Aug	15:38	15:40	W/M (2)	15:09 (29)	Good long infrasound precursor
30 Aug	17:12	17:25	W/M (1)	17:02 (10)	Clear precursor despite relatively small sized event
02 Sep	14:24	14:00	W/M (2/3)	13:56 (28)	Long-duration infrasound precursor with pulses
05 Sep	17:40	17:45	M/S (2/3)	17:07 (33)	Excellent infrasound precursor and energetic flow front
12 Sep	15:35*	17:24	W/M (1)	15:23 (16)	Example of a *correntada,* which is a long gradually increasing flow without obvious surging onset
13 Sep	15:09	15:20	W/M (2/3)	14:36 (33)	Well-defined long duration precursor; INSIVUMEH suggests possible lahar in another drainage at same time
14 Sep	17:29	17:35	W/M (3)	17:04 (25)	Very strong initial flow front
27 Sep	16:53	17:00	W/M	16:36 (17)	Second event comes later
29 Sep	14:49	15:02	W/M (2/3)	14:28 (21)	Possible precursor obscured by thunder
30 Sep	17:08	17:20	W/M (2)	16:54 (14)	Similar to previous event; noisy thunder
01 Oct	17:01	17:05	W/M (3)	16:34 (27)	Excellent obvious infrasound precursor and strong flow front
02 Oct	17:17	18:00	W/M (1/2)	Na	Noisy thunder; no noticeable precursor
13 Oct	17:55	17:55	M/S (2/3)	Na	Noisy thunder; flow front passes in failing light

Column 4 INSIVUMEH size (video size) refers to INSIVUMEH reports, which rank lahars based upon seismic amplitudes as either (W)eak—debil, (M)oderate—moderado, or (S)trong—fuerte. Parenthetical number refers to qualitative evaluation of flow front size as seen in time lapse footage where (1) is smallest and (3) is largest. An asterisk (*) in Column 2, video surge detection, indicates flows for which considerable flow was already evident when the flow head arrives, i.e., correntadas. Time lapse videos of these lahars and time-synchronized infrasound are viewable at https://www.youtube.com/watch?v=wfzVMHiPI2o and described in Additional Information.

Although featured lahar sizes are variable and infrasound signal quality was affected by a wide range of noise (thunder being most pernicious), decent lahar precursors were identifiable in 18 of 22 events (see Table 2 and videos linked at https://www.youtube.com/watch?v=wfzVMHiPI2o in Additional Information). For those four events occurring on August 12, August 24, October 2, and October 13, clear precursors were not evident; we consider that infrasound noise may have been especially high and/or the flow head might not have arrived as a significant surge. Lahars that increase gradually and/or begin as *correntadas* tend not to present as highly coherent upstream sources because the sound adjacent to the station from low-level fluvial activity is ongoing and/or may dominate.

The observation of strong, easy-to-identify infrasound precursors for flows that begin with a surge is auspicious for monitoring purposes. These are the flows that are potentially the most damaging because they have the capability to arrive suddenly and surprisingly with an energetic lahar head. Some of the larger 2022 lahars exhibit infrasound precursors as long as 30 min detected at FG14. This length of time could avail itself to timely warnings once automated systems are developed. We suspect that in these cases the detected lahar sounds are being picked up in a zone as far away as 7 km above the infrasound array and in the vicinity of 7 to 8 km channel distance. Detections in these locations, if relayed as social media alerts, could allow timely evacuation of downstream locations where vehicles and/or people typically cross the channel.

We propose that infrasound monitoring is an important complement to co-located seismic monitoring. In the case of the Ceniza drainage INSIVUMEH uses ground vibrational tremor amplitudes to successfully issue alerts of a lahar in progress and these are produced with a high degree of reliability and generally less than 10 to 20 min after the lahar has passed FG14. Seismic sensors situated higher up in Ceniza would be capable of definitively detecting earlier lahar movement—at around the same time that downstream infrasound arrays begin to identify flows—however their situation and maintenance high up on the volcano is logistically complicated and costly.

Lahars similar in size to those shown in Table 3 have been frequently recurring at Fuego since the current cycle of activity began in 1999. Although these mudflows are much smaller than the primary lahars at Nevado del Ruiz^1^, Mount St. Helens^3^, or Villarrica^22^, they represent a persistent seasonal hazard for the many road crossings (fords) at active channels, where incidents of vehicles caught by lahars have happened over the last few years. Advanced notice of lahars approaching the crossing points could allow timely closing of the road traffic to avoid such incidents in the future. An important question then becomes how and where to configure seismo-acoustic sensors in the most efficient topology given finite hardware resources and the often-difficult access and maintenance of upstream locales.

## Concluding thoughts

The capabilities of infrasound as a lahar monitoring tool are evident for infrasound sensors used in array configuration with as few as two sensors. Application of cross-correlation analysis for sensors spaced 30 to 50 m apart makes it possible to discriminate noise and detect signals originating from upstream sources that combine constructively and reach the sensor array with a consistent direction. Lahar sound distributed along a straight river path can thus ‘pile up’ and be used to identify the onset of subtle or emergent infrasound. Infrasound arrays situated a distance away from the lahar flow path may not be as sensitive to detection of low signal-to-noise infrasound, but they do have the benefit of detecting flow movement reflected by shifting cross correlation time lags and a systematic change in the direction of incident sound; in order to track lahar movements situation of infrasound arrays set back from the lahar channel is thus recommended. Although the lahars featured in this study are small compared with the large primary lahars of Armero (Colombia) in 1985^1^, Mount St. Helens (US) in 1980^21^, or Villarrica (Chile) in 2015^22^, the infrasound radiation is still sufficient to identify an approaching flow even in the presence of substantial background noise. This work contributes to the growing field of environmental seismology^42^, which has a focus on gravity-driven mass movement and is increasingly making use of both ground-propagating seismic as well as atmosphere-propagating infrasound wavefields. Lahar seismo-infrasound is an example of a geophysical discipline where scientific study and hazard monitoring objectives are closely intertwined.

## Methods

### Array analysis

Data from three-element infrasound mini-arrays were analyzed at stations IO, IC, and IF (where three of the four permanent microphones were utilized). Data were recorded at IO and IC with infraBSU version #2 microphones and DiGOS Datacube 24-bit dataloggers, and with Chapparal microphones and radio telemetry at IF. All data were recorded at 200 Hz (IO and IC) or upsampled from 50 to 200 Hz (at IF) prior to application of a high pass, two-pole 1 Hz filter. More information on the technical specifications and operations of the infraBSU infrasonic microphones can be found in^44^**.** Precision relative locations of infrasound sensors is important for array analysis and positional information at IO and IC were surveyed using mapping grade Emlid Reach RS2 GPS. All infrasound waveform data were recorded with a common GPS-enabled clock, which is a feature of the respective digitization systems. The timing of the stand-alone camera video is accurate to within 1 s confirmed by regularly filming of a cell phone digital clock.

Time delays between sound arriving at microphones can be used to extract sound location information from each array (e.g., Fig. 3a). In each time window where correlated time signal is identified the peak correlation lag times are calculated and used to determine the most likely location for the incident sound. From the array data at IO the locations of possible sources were inferred based upon the expected lag times for sources confined to Ceniza’s channel, the known array sensor positions, and an assumed speed of 343 m/s. The channel path of candidate source locations was derived from 8-m resolution digital topography produced after the June 3, 2018 paroxysm by the UN-SPIDER project. The channel was drawn from the summit using a steepest gradient descent in to the Ceniza drainage. Channel distances shown in Fig. 1b are relative to the summit and mapped to time lags at IO such that each discrete time lag corresponds to a distance along Ceniza (Fig. 3a). Forward azimuths for these sources are indicated parenthetically. Assuming a lahar in Ceniza it is thus possible to locate a dominant source with only one pair of sensors, but multiple sensor pairs can be used to corroborate locations. Uncertainty in channel location may be calculated as positional uncertainty for a given cross-correlation time lag variability. We compute an uncertainty ε in terms of km/sample. The inverse, or positional precision, is indicated in Fig. 3c and shows best precision for lahars located between 8 and 11 km. Sensor pairs for channels 3 and 2 and 1 and 3 have better precision than sensor pair 2 and 1 because their orientation is sub-parallel to the drainage. This technique for locating position in Ceniza is the same as that utilized for a lahar occurring at Villarrica Volcano (Chile) in 2015^22^**.**

Thunder and volcanic vent activity, considered as noise in this study, are identified as short-duration highly-correlated transient events lasting less than 1 min (e.g., Figs. 1b, 4e,f). Nearly all lahar infrasound events from Table 3 have varying amounts of thunder and volcano infrasound, which are identified by calculating a ratio of short term (5 s) to long-term (60 s) average power. We identify and label these transients when the short-term long-term ratio exceeds a threshold value of 3 for events separated by 30 s or more. These values are arbitrary, but work well to identify discrete events, which look to be eruptions or thunder during visual review. We then classify thunder and eruption infrasound based upon their distinct spectral content in the 1 to 100 Hz band. Those events with 50% of their energy below 20 Hz are found to be volcanic in origin, while those with 50% of energy above 20 Hz are categorized as thunder. During the six-hour record of infrasound activity on August 17 transient detections included 30 eruption events and 65 thunder signals (Fig. 1b).

## Supplementary Information

Supplementary Information.

## Data Availability

Waveform data for the 17 August event are provided as MATLAB files at Boise State University’s Infrasound Data Repository (https://scholarworks.boisestate.edu/infrasound_data/). These data, along with a README text file, are archived as *Dataset for Infrasound Early Warning Detections for Lahars* with a dedicated https://doi.org/10.18122/infrasound_data.10.boisestate. Time lapse video and camera footage of the featured lahars are at this repository following publication. They are also posted as YouTube videos (https://www.youtube.com/watch?v=wfzVMHiPI2o and https://www.youtube.com/watch?v=O9lJT52MVik).
