# Supplementary material for: Infrasound detection of approaching lahars
Source: Sci Rep. 2023 Apr 20;13:6476. doi: 10.1038/s41598-023-32109-2 (PMC10119103; doi:10.1038/s41598-023-32109-2)
Supplement: Supplementary file 1 — Supplementary Information. [file 41598_2023_32109_MOESM1_ESM.docx]

**Additional Information**

**Supplementary animations.**

The two animations described below are provided as supplements linked as YouTube videos and are to be posted at the data repository (see Data Availability above).

*Ceniza_22Lahars_2022.mp4* <https://www.youtube.com/watch?v=wfzVMHiPI2o> . This 32-minute video is a collection of 22 lahar events occurring in 2022 and featured in Table 3. The video is created from 40-s-interval time lapse footage from CF (INSIVUMEH’s station FG14) along with time-synchronized infrasound and seismic power time series from CF and sF. Cross-correlation analysis shows the evolution of correlated signal as described in the text. Lahar precursors are defined as correlated signal occurring prior to the (visual) arrival of a flow pulse arriving at camera CF and are the same values as shown in Table 3. Video and audio have been sped up by a factor of 60 such that 1 minute is 1 second and 1 to 20 Hz infrasound becomes audible 60 to 1200 Hz audible sound.

*Ceniza_HD_Animation_Aug17* <https://www.youtube.com/watch?v=O9lJT52MVik> . This video is a ten-minute sequence from 17 August 2022 depicting the passage of the featured lahar between camera CB and CR. Video and waveform data are time synchronized and shown in real-time. Map shows the 2 km transect and approximate location of lahar position over time. Audio is provided from camera CB (until 8 minutes in to the video) and then from camera CR (after 8 minutes). The lahar arrives at CB at ~1:30 and at CR at ~8:15 in this video.
